# Neutrophil extracellular traps in diabetic wound healing: mechanisms, pathological roles, and therapeutic implications

**DOI:** 10.1093/burnst/tkag041

**Published:** 2026-06-11

**Authors:** Zhihan Hu, Yunwei Wang, Jiatong Wang, Guangtong Cao, Xiaoyu Di, Zongjiang Yao, Gang Wang, Yuchen Kang, Ruomei Zhao, Yi Liu

**Affiliations:** Department of Burn Plastic and Wound Repair Surgery, Lanzhou University Second Hospital, No. 82 Cuiyingmen, Lanzhou, Gansu 730030, China; Key Laboratory of Stem Cells and Gene Drugs, The 940 Hospital of the Joint Logistics Support Force of the Chinese People’s Liberation Army, No. 333, Nanbinhe Road, Qilihe District, Lanzhou, Gansu 730030, China; Department of Burn Plastic and Wound Repair Surgery, Lanzhou University Second Hospital, No. 82 Cuiyingmen, Lanzhou, Gansu 730030, China; Key Laboratory of Stem Cells and Gene Drugs, The 940 Hospital of the Joint Logistics Support Force of the Chinese People’s Liberation Army, No. 333, Nanbinhe Road, Qilihe District, Lanzhou, Gansu 730030, China; Department of Burns and Cutaneous Surgery, Xijing Hospital, Fourth Military Medical University, 127 West Changle Road, Xi’an, Shaanxi 710032, China; Department of Burn Plastic and Wound Repair Surgery, Lanzhou University Second Hospital, No. 82 Cuiyingmen, Lanzhou, Gansu 730030, China; Department of Pharmacology, School of Pharmacy, Air Force Medical University, No. 169 Changle West Road, Xincheng District, Xi’an, Shaanxi 710032, China; Department of Burn Plastic and Wound Repair Surgery, Lanzhou University Second Hospital, No. 82 Cuiyingmen, Lanzhou, Gansu 730030, China; Key Laboratory of Stem Cells and Gene Drugs, The 940 Hospital of the Joint Logistics Support Force of the Chinese People’s Liberation Army, No. 333, Nanbinhe Road, Qilihe District, Lanzhou, Gansu 730030, China; Department of Burn Plastic and Wound Repair Surgery, Lanzhou University Second Hospital, No. 82 Cuiyingmen, Lanzhou, Gansu 730030, China; Key Laboratory of Stem Cells and Gene Drugs, The 940 Hospital of the Joint Logistics Support Force of the Chinese People’s Liberation Army, No. 333, Nanbinhe Road, Qilihe District, Lanzhou, Gansu 730030, China; Department of Burn Plastic and Wound Repair Surgery, Lanzhou University Second Hospital, No. 82 Cuiyingmen, Lanzhou, Gansu 730030, China; Department of Burn Plastic and Wound Repair Surgery, Lanzhou University Second Hospital, No. 82 Cuiyingmen, Lanzhou, Gansu 730030, China; Department of Burn Plastic and Wound Repair Surgery, Lanzhou University Second Hospital, No. 82 Cuiyingmen, Lanzhou, Gansu 730030, China; Key Laboratory of Stem Cells and Gene Drugs, The 940 Hospital of the Joint Logistics Support Force of the Chinese People’s Liberation Army, No. 333, Nanbinhe Road, Qilihe District, Lanzhou, Gansu 730030, China; Department of Burn Plastic and Wound Repair Surgery, Lanzhou University Second Hospital, No. 82 Cuiyingmen, Lanzhou, Gansu 730030, China; Key Laboratory of Stem Cells and Gene Drugs, The 940 Hospital of the Joint Logistics Support Force of the Chinese People’s Liberation Army, No. 333, Nanbinhe Road, Qilihe District, Lanzhou, Gansu 730030, China; Department of Burn Plastic and Wound Repair Surgery, Lanzhou University Second Hospital, No. 82 Cuiyingmen, Lanzhou, Gansu 730030, China

**Keywords:** Neutrophil extracellular traps, Neutrophil extracellular trap formation, Diabetic wounds, Chronic inflammation, Wound healing

## Abstract

Diabetic wound healing is a common yet challenging problem in clinical practice that involves complex pathophysiological processes and frequently progresses to chronic nonhealing wounds, imposing substantial burdens on both healthcare systems and patients while markedly decreasing quality of life. Persistent inflammatory responses represent a fundamental pathological feature of this condition. Accumulating evidence highlights the key role of neutrophil extracellular traps (NETs) in the chronic inflammatory response characteristic of diabetic wounds. As components of the innate immune system, NETs play pivotal roles in both host defence and tissue repair. Neutrophil extracellular trap formation (NETosis) is currently classified as vital NETosis or lytic NETosis. In this review, we synthesize existing evidence on the mechanistic heterogeneity of NETosis and further refine lytic NETosis subtypes on the basis of distinct molecular mechanisms and temporal dynamics—namely, NADPH oxidase 2 (NOX2)-dependent classical lytic NETosis and mitochondrial reactive oxygen species–driven rapid lytic NETosis. We also highlight the functional outcomes of NETs in response to specific stimuli within the diabetic wound milieu. Therapeutic strategies targeting NET formation, degradation, or neutralization have shown considerable promise in preclinical studies; however, their clinical translation will require standardized biomarkers for NET quantification, localized delivery approaches to minimize systemic immunosuppression, and biomarker-guided frameworks to balance the risk of infection against healing benefits.

## Highlights

- Lytic neutrophil extracellular trap formation (NETosis) can be refined into NADPH oxidase 2 (NOX2)-dependent classical and mitochondrial ROS (mtROS)-driven rapid subtypes.
- Hyperglycaemia primes neutrophils for excessive neutrophil extracellular trap (NET) release *via* metabolic reprogramming.
- NETs exhibit concentration-dependent dual roles in diabetic wound healing.
- Targeting NET formation/degradation shows promise but requires biomarker-guided local delivery.
- Multimodal strategies combining NET inhibition and antimicrobial therapy warrant exploration.

## Background

Diabetes mellitus is one of the most pressing global health challenges of the twenty-first century. The major clinical burden of this metabolic disorder stems from its long-term complications, which manifests in the skin and soft tissues as an increased predisposition of diabetic wounds to progress to chronic, nonhealing ulcers. The pathophysiology of diabetic wound healing is multifactorial, driven by the complex interplay among persistent hyperglycaemia, peripheral neuropathy, microvascular dysfunction, and local immune microenvironment dysregulation, and is characterized by a prolonged inflammatory phase, impaired angiogenesis, and defective re-epithelialization. Thus, diabetic wounds impose a substantial burden on healthcare systems and patients’ families worldwide, and these chronic lesions frequently progress to amputation in severe cases, severely undermining patients’ quality of life.

The current clinical management of diabetic wounds can be broadly categorized into conservative treatment and surgical intervention. Conservative strategies focus predominantly on glycaemic control, regular wound cleansing, and dressing changes, with the goal of maintaining a moist healing environment. When conservative measures prove insufficient, surgical approaches are employed, including thorough debridement of necrotic tissue, and for wounds that cannot be closed directly, split-thickness skin grafting or flap surgery are used for reconstruction. However, these conventional modalities share a fundamental limitation: they target the manifestations of impaired healing rather than the underlying pathophysiological mechanisms that sustain the chronic, nonhealing state. Consequently, clinical outcomes remain unsatisfactory and highly variable. Many patients endure prolonged treatment courses with frequent dressing changes but fail to achieve infection control or wound closure. Even after surgical intervention, wounds may exhibit delayed healing at the site of repair, experience recurrent breakdown within weeks or months, or, in some cases, progressive enlargement despite apparent initial improvement. Although emerging regenerative therapies—including topical growth factors and bioengineered skin substitutes—have shown promise in certain clinical scenarios, their efficacy is often compromised by the intrinsic characteristics of the diabetic wound microenvironment, particularly the persistent inflammatory milieu and increased susceptibility to microbial colonization. These therapeutic shortcomings underscore a critical reality: without elucidating the precise immune drivers of wound chronicity and identifying novel molecular targets capable of resetting the dysregulated healing trajectory, substantial improvements in clinical outcomes will remain limited. A deeper understanding of the immunopathological processes that distinguish diabetic wounds from normally healing wounds is therefore urgently needed.

In this context, neutrophil extracellular traps (NETs) have emerged as key mediators of the chronic inflammatory state characteristic of diabetic wounds. First described in 2004 as an antimicrobial defence mechanism, NETs are reticular structures composed of decondensed chromatin and cytotoxic granule proteins that are released by activated neutrophils through a distinct form of programmed cell death termed neutrophil extracellular trap formation (NETosis). It is now recognized that NETs exert concentration-dependent and context-specific effects on tissue repair. Under physiological conditions, low levels of NETs may promote wound healing by modulating the activity of reparative cells. In the diabetic microenvironment, however, hyperglycaemia, oxidative stress, and advanced-glycation end products drive excessive NET formation, and such pathological increases in NET concentrations drive chronic inflammation and tissue damage. This duality renders NETs promising therapeutic targets. Moreover, the current classification of NETosis subtypes remains largely confined to the presence or absence of cell membrane lysis, but a more refined distinction based on specific molecular mechanisms and the temporal dynamics of NET release is essential to clarify the predominant forms of NETosis that occur in diabetic wounds and to further advance targeted intervention strategies.

This review aims to summarize the current knowledge on the mechanisms of NETosis, with particular attention given to the comparative research between NOX2-dependent lytic NETosis and mitochondrial reactive oxygen species (ROS)–driven rapid lytic NETosis, to further refine the classification of lytic NETosis subtypes according to their triggering mechanisms and temporal profiles. We first delineate the molecular mechanisms by which hyperglycaemia and metabolic stress drive excessive NET release from neutrophils and present existing evidence on the formation, regulation, and functional heterogeneity of NETs within the diabetic wound microenvironment. We subsequently analyse the dual roles of NETs, namely, their beneficial effects at physiological concentrations versus their detrimental effects upon pathological accumulation. Finally, we evaluate emerging therapeutic strategies targeting NET formation, degradation, or neutralization and discuss the challenges of clinical translation and the necessity of biomarker-guided interventions. By integrating mechanistic insights with a translational perspective, this review highlights that NETs are not mere bystanders but instead central orchestrators of impaired diabetic wound healing, thereby providing a theoretical rationale for their targeted modulation as a strategy to promote wound repair.

To ensure methodological transparency and reproducibility, we conducted a systematic literature search using primarily PubMed supplemented by Web of Science and Google Scholar. The search period spanned from 2004, when NETs were first described, to 2025. Key search terms included ‘neutrophil extracellular traps’, ‘NETosis’, ‘diabetic wounds’, ‘wound healing’, and their combinations. We included peer-reviewed original research articles and reviews that addressed the mechanisms, pathological roles, and therapeutic implications of NETs in diabetic wound healing. This review synthesizes the available evidence with a focus on the mechanistic heterogeneity of NETosis and its translational relevance.

## Review

### Definition, composition, and detection methods of NETs

NETs are extracellular fibrillar structures composed of disaggregated chromatin studded with various cytoplasmic proteins and are actively released by activated neutrophils. NET release involves a series of unique cellular events and is therefore described as a new form of programmed cell death distinct from apoptosis, termed NETosis [1–3]. The reticular DNA in NETs is derived predominantly from the cell nucleus, although the presence of mitochondrial DNA has been detected in some cases [4]. The reticular structure of NETs, in conjunction with the various antimicrobial proteins modified on their surface, such as histones, cathepsin G, defensins, antimicrobial peptides (including LL37), and peptidoglycan-binding proteins with bactericidal activity, enables them to capture and kill pathogens, thereby exerting immune functions [5–8]. In addition to neutrophils, macrophages, mast cells, eosinophils, and basophils can produce extracellular traps (ETs) [9–18].

As part of the innate immune response, NETosis can be triggered by various pathogen-associated molecular patterns (PAMPs), such as bacteria [6, 19, 20], fungi [21–26], and viruses [27], as well as by damage-associated molecular patterns (DAMPs), including advanced glycation end products (AGEs), high-mobility group box 1 (HMGB1), autoantibodies, immune complexes, complement components, and platelet activation signals [3, 28–30]. The presence of free DNA, MPO-DNA complexes, and citrullinated histones (such as H3Cit) has been recognized as a hallmark of NETs [31].

### Types of NETosis

As demonstrated in the literature, there are two recognized mechanisms of NET release, which are classified on the basis of neutrophil fate after they release NETs [2, 32]. The two forms of NETosis are distinguished by the presence or absence of cell membrane lysis. Lytic NETosis is characterized by lysis of the neutrophil cell membrane, whereas vital NETosis is characterized by retention of an intact cell membrane and phagocytic function. By integrating the recent evidence on NETosis mechanisms and the temporal dynamics of NET release, lytic NETosis can be further subdivided into classical lytic NETosis and rapid lytic NETosis. The former is characterized by ROS bursts dependent on NADPH oxidase 2 (NOX2) activation, whereas the latter is driven by mitochondrial ROS production (Table 1).

**Table 1:** Characteristics of the three types of NETosis

	Lytic NETosis	Rapid lytic NETosis	Vital NETosis
Neutrophil Fate	Cell death (membrane lysis)	Cell death (membrane lysis)	Viable cells (membrane integrity)
Inducing Factor	Bacteria [1, 6, 19, 33, 34], fungi [21–26], AGEs [35], HMGB1 [36], PMA, autoantibodies, etc.	Bacteria, fungi, chemokines, high concentrations of C5a [37, 38], etc.	Bacteria [39], ANCA immune complexes [40–43], C3a, low concentrations of C5a [39], platelet activation signals [44] (P-selectin), etc.
Pattern Recognition Receptors (PRRs)	TLR-MyD88 [39] (TLR1/2/4/5/6), RAGE [35], Dectin-1 [21–26]	TLR-TRIF [45] (TLR3/4), C5aR1 [37, 38]	C3aR [39], TLR2, C5aR1, FcγR [40–42]
ROS Dependency	NOX2	mtROS	mtROS
Chromatin Disaggregation	Dependent on PAD4 enzyme-mediated CitH3 and synergises with NE to promote chromatin disassembly	Dependent on PAD4 enzyme-mediated CitH3 and synergises with NE; HMGB1 binding to DNA may assist with chromatin decondensation	mtROS or NE directly cleave histones, causing chromatin disassembly
Gasdermin D	Nonessential	High expression levels of caspase-1 or caspase-11 result in high-intensity cleavage of GSDMD, leading to widespread cell membrane pore formation and the promotion of membrane lysis	Low expression levels of caspase-1 cleave GSDMD locally in the cell membrane
**Chromatin Release**	Nuclear membrane lysis	Nuclear membrane lysis	Intranuclear vesicle transport
Process Duration	Slow (2–4 h)	Extremely fast (<30 min)	Fast (<1 h)
Related Diseases	Bacterial infection, chronic inflammation (diabetic wounds), autoimmune diseases	Acute trauma, tissue necrosis, acute bacterial infection, fungal infection	Thrombosis, sepsis, AAV

*AAV* ANCA-associated vasculitis, *AGEs* advanced-glycation end products, *ANCA* anti-neutrophil cytoplasmic antibody, *C3aR* complement C3a receptor, *C5aR1* complement C5a receptor 1, *CitH3* citrullinated histone H3, *Dectin-1* dendritic cell–associated C-type lectin-1, *FcγR* Fc gamma receptor, *GSDMD* gasdermin D, *HMGB1* high mobility group box 1, *mtROS* mitochondrial reactive oxygen species, *MyD88* myeloid differentiation primary response 88, *NE* neutrophil elastase, *NETosis* neutrophil extracellular trap formation, *NOX2* NADPH oxidase 2, *PAD4* peptidyl arginine deiminase 4, *PMA* phorbol 12-myristate 13-acetate, *PRRs* pattern recognition receptors, *RAGE* receptor for advanced-glycation end products, *TLR* toll-like receptor

#### Lytic NETosis (suicidal NETosis)

**Figure 1:**
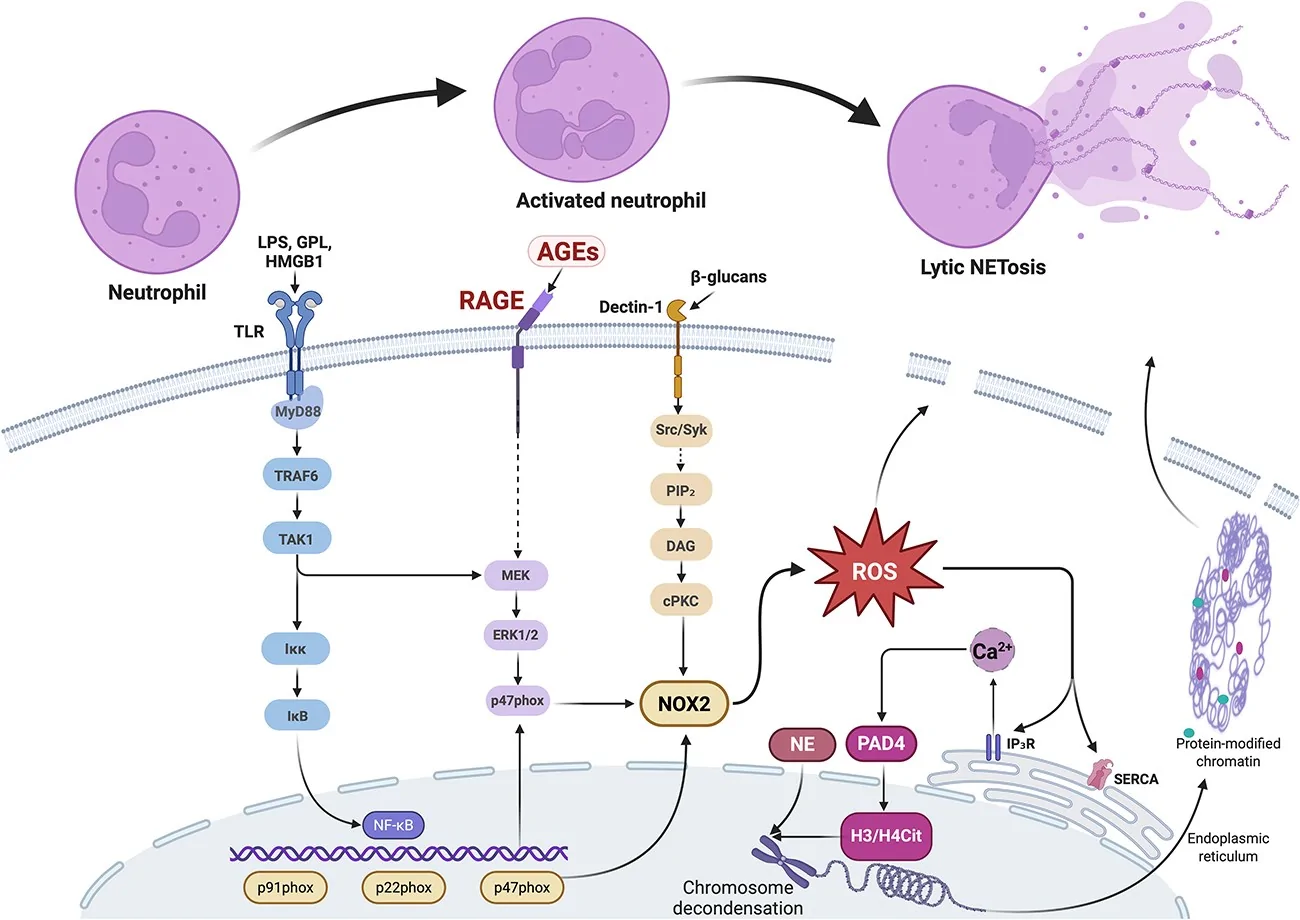
The mechanism schematic of Lytic NETosis. Classic lytic NETosis depends on the activation of receptors, including MyD88-dependent TLRs, RAGE, and Dectin-1, leading to the assembly and activation of the NOX2 complex and the subsequent induction of an intracellular ROS burst. NOX2-derived ROS oxidize specific cysteine residues on IP₃R located on the endoplasmic reticulum, sensitizing it to IP₃ and promoting Ca^2+^ release; moreover, ROS inhibit SERCA pumps through oxidation, preventing Ca^2+^ re-uptake. The resulting Ca^2+^ surge activates the Ca^2+^-dependent enzyme PAD4. Activated PAD4 catalyses the citrullination of arginine residues on histone H3/H4 in the nucleus, resulting in increased nuclear membrane permeability. NE combines with MPO to collectively promote chromatin decondensation. Created in https://BioRender.com. *AGEs* advanced-glycation end products, *DAG* diacylglycerol, *ERK1/2* extracellular signal–regulated kinase 1/2, *HMGB1* high mobility group box 1, *H3/H4Cit* citrullinated histone H3/H4, *IKK* IκB kinase, *IκB* inhibitor of κB, *IP3R* inositol 1,4,5-trisphosphate receptor, *LPS* lipopolysaccharide, *MEK* mitogen-activated protein kinase kinase, *MPO* myeloperoxidase, *MyD88* myeloid differentiation primary response 88, *NE* neutrophil elastase, *NF-κB* nuclear factor kappa B, *NOX2* NADPH oxidase 2, *PAD4* peptidyl arginine deiminase 4, *PIP2* phosphatidylinositol 4,5-bisphosphate, *RAGE* receptor for advanced-glycation end products, *ROS* reactive oxygen species, *SERCA* sarcoplasmic/endoplasmic reticulum Ca2+-ATPase, *Syk* spleen tyrosine kinase, *TAK1* transforming growth factor-β-activated kinase 1, *TLR* toll-like receptor, *TRAF6* TNF receptor-associated factor 6

The classical form of NETosis, termed lytic NETosis, is triggered by PAMPs and DAMPs such as bacteria, AGEs, autoantibodies, or phorbol esters [19] (Figure 1). The most common route involves toll-like receptor (TLR) family activation [46, 47], and most pathways depend on the TLR-MyD88 axis [39]. For example, LPS from gram-negative bacteria engages TLR4, peptidoglycans from gram-positive bacteria signals *via* TLR1/2 or TLR2/6, HMGB1 binds to TLR2/4, flagellin acts through TLR5, and viral ssRNA is recognized by TLR7/8 [1, 6, 19, 33, 34, 36, 48, 49].

Upon TLR activation, MyD88 recruits IRAKs and TNF receptor–associated factor 6 (TRAF6), which then phosphorylate TGF-β-activated kinase 1 (TAK1) [46, 47]. TAK1 activation simultaneously triggers the mitogen-activated protein kinase/extracellular signal-regulated kinase (MAPK/ERK) pathway and the NF-κB pathway [50]. The MAPK/ERK arm upregulates proteins needed for NOX2 assembly (e.g. p47phox), whereas NF-κB directly drives NOX2 gene transcription [28]. Accordingly, ERK inhibitors only partially reduce NET release [51], whereas NF-κB inhibitors more strongly suppress NOX2 expression and ROS generation [52, 53]. NOX2 activation promotes a burst of ROS. Additionally, β-glucans from fungi such as *Candida albicans* can stimulate ROS production *via* the Dectin-1 receptor pathway (Src/Syk/PLCγ/PIP₂/DAG/cPKC/NOX2) [21–26, 54].

These NOX2-derived ROS oxidize specific cysteine residues on IP₃ receptors (IP₃Rs), sensitizing them to IP₃ and promoting Ca^2+^ release from the endoplasmic reticulum [55]. Moreover, ROS inhibit SERCA pumps through oxidation, preventing Ca^2+^ re-uptake and causing Ca^2+^ leakage from the ER [56]. The resulting Ca^2+^ surge activates the Ca^2+^-dependent enzyme PAD4 [57]. Activated PAD4 catalyses the citrullination of histone H3/H4 (H3Cit), leading to chromatin decondensation [58, 59]. Furthermore, NE and myeloperoxidase (MPO) are released from azurophilic granules and translocate to the nucleus. NE degrades histone H1 and core histones (especially H4), disrupting the nucleosome structure in synergy with MPO [28, 60]. Structural research has shown that MPO dimers disassemble nucleosomes by binding the H2A-H2B acidic patch *via* arginine anchors (Arg473/Arg653), whereas MPO monomers bind mature NETs without disassembly *via* a chromatin remodelling function independent of the enzymatic activity of MPO [61]. In addition, the temporal relationship between PAD4-mediated citrullination and NE/MPO-mediated histone cleavage is still under debate; under certain stimuli, NE can translocate to the nucleus without PAD4, and PAD4-independent NETosis pathways exist [notably in vital NETosis, where mitochondrial ROS (mtROS) or NE may directly cleave histones] [39, 62]. Thus, the interdependence of PAD4 and NE is likely stimulus-specific. Finally, decondensed chromatin studded with granule and cytoplasmic proteins is released as mature NETs through programmed cell membrane rupture [28]. The time from phorbol 12-myristate 13-acetate (PMA) stimulation to NET release *ex vivo* is ~2–4 h, depending on the stimulus. Many *in vitro* experiments have shown that PAD4 inhibitors effectively block lytic NETosis [63–71].

#### Rapid lytic NETosis

**Figure 2:**
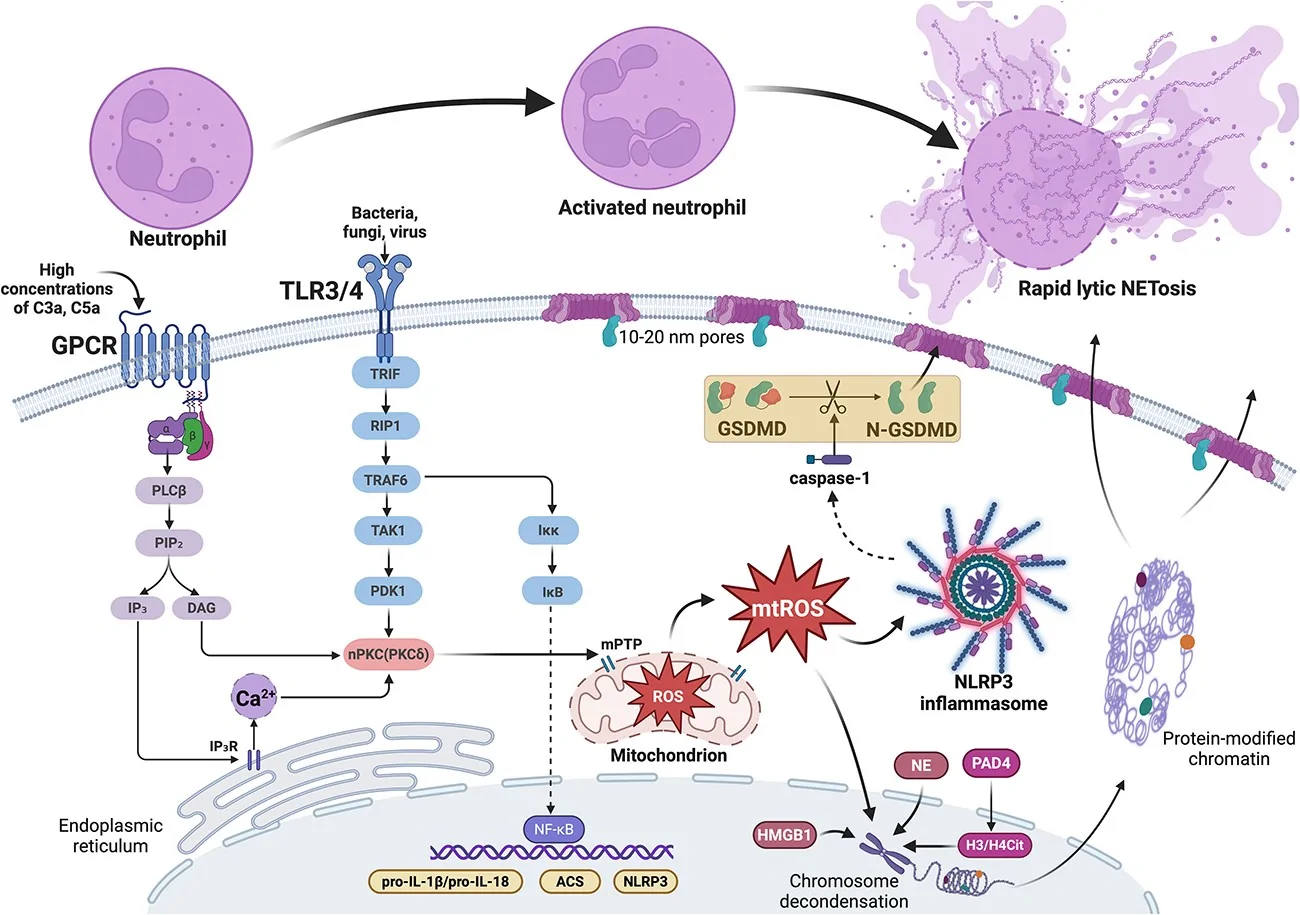
The mechanism schematic of Rapid lytic NETosis. Rapid lytic NETosis does not necessarily depend on NOX2 but instead relies on TRIF-dependent TLR receptor-induced nPKC activation or activation by high concentrations of complement receptors, which causes a rapid increase in the cytosolic Ca^2+^ concentration owing to its release from endoplasmic reticulum stores, thereby activating nPKC. Activated nPKC induces the collapse of the mitochondrial membrane potential, subsequently triggering mtROS production. Furthermore, the TLR-TRIF pathway induces NLRP3 inflammasome activation, in which caspase-1 robustly cleaves and activates GSDMD, thereby forming extensive pores in the plasma membrane and ultimately leading to neutrophil lysis and NET release. Additionally, Ca^2+^ signalling can increase PAD4 enzyme-catalysed histone citrullination and the binding of HMGB1 to DNA, synergizing with mtROS to promote chromatin decondensation. Created in https://BioRender.com. *ACS* acetylsalicylic acid, *C3a/C5a* complement components 3a/5a, *DAG* diacylglycerol, GPCR G-protein-coupled receptor, *GSDMD* gasdermin D, *H3/H4Cit* citrullinated histone H3/H4, *HMGB1* high mobility group box 1, *IKK* IκB kinase, *IκB* inhibitor of κB, *IP₃* inositol 1,4,5-trisphosphate, *IP₃R* inositol 1,4,5-trisphosphate receptor, mPTP mitochondrial permeability transition pore, *mtROS* mitochondrial reactive oxygen species, *NE* neutrophil elastase, *NF-κB* nuclear factor kappa B, *N-GSDMD* N-terminal gasdermin D, *NLRP3* NOD-like receptor family pyrin domain containing 3, *PAD4* peptidyl arginine deiminase 4, *PDK1* 3-phosphoinositide-dependent protein kinase-1, *PIP₂* phosphatidylinositol 4,5-bisphosphate, *PLCβ* phospholipase C beta, *IL* interleukin, *RIP1* receptor-interacting protein 1, *ROS* reactive oxygen species, *TAK1* transforming growth factor-β-activated kinase 1, *TLR3/4* toll-like receptor 3/4, *TRAF6* TNF receptor–associated factor 6, *nPKC(PKCδ)* novel protein kinase C (protein kinase C delta)

Both rapid and classical lytic NETosis result in neutrophil death, but a key distinction is the activation of gasdermin D (GSDMD) in the former process, which causes rapid membrane disintegration and NET release within minutes to hours [20, 72, 73] (Figure 2). This process resembles pyroptosis [74–76]. The proposed mechanism involves high levels of caspase-1-cleaved GSDMD or the direct noninflammasome-dependent caspase-11 cleavage of GSDMD, leading to oligomerization of its N-terminal domain (GSDMD-NT) and the formation of 10–20 nm pores in the cell membrane [72, 76–78]. An alternative view suggests that during NETosis, serine proteases such as NE can cleave and activate GSDMD independent of caspases, generating N-terminal domains that form membrane pores [79].

Rapid lytic NETosis does not necessarily require a NOX2-driven ROS burst; instead, it can be triggered by nPKC (PKCδ)/Ca^2+^-induced mtROS production [20, 63, 73, 80, 81]. For instance, the GPCR C5aR1 responds to high levels of complement C5a or chemokines (e.g. IL-8) *via* classical G protein signalling, directly activating calcium channels and increasing intracellular Ca^2+^ levels. Activated PKCδ then induces the opening of the mitochondrial permeability transition pore (mPTP), leading to the collapse of the mitochondrial membrane potential and triggering the production of mtROS [37, 38, 54]. mtROS, in turn, activates the NLRP3 inflammasome, which is followed by GSDMD activation [38, 72, 76, 82]; indeed, the NLRP3 inflammasome supports NETosis under sterile conditions by promoting nuclear envelopment and plasma membrane breakdown [83]. Moreover, TRIF-dependent TLRs (e.g. TLR3/4) can trigger mtROS generation *via* the RIP1/TRAF6/nPKC axis, and TRIF signalling can prime NLRP3 inflammasome activation [45, 84–86]. The precise mechanism of chromatin decondensation in rapid lytic NETosis has not yet been elucidated, but calcium signalling may facilitate this process by increasing PAD4-catalysed histone citrullination (H3Cit) and the binding of HMGB1 to DNA.

#### Vital NETosis

**Figure 3:**
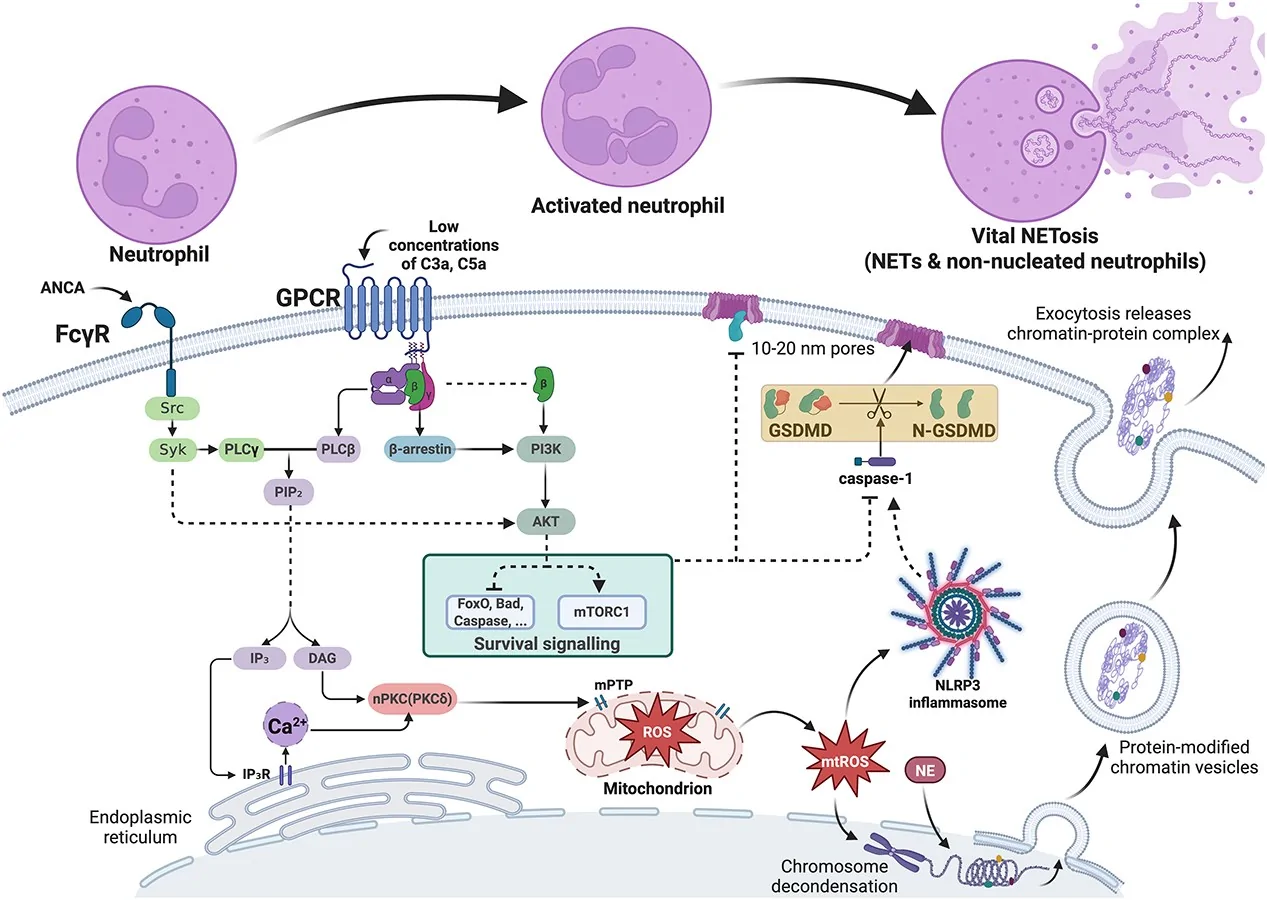
The mechanism schematic of Vital NETosis. Vital NETosis depends entirely on the activation of complement receptors, FcγR, and other receptors that induce Ca^2+^ release from endoplasmic reticulum stores *via* IP3, leading to increased cytosolic Ca^2+^ concentrations and the subsequently activation of nPKC. This results in the collapse of the mitochondrial membrane potential and triggers mtROS production. The distinction from lytic NETosis may lie in the activation of the PI3K/AKT pathway *via* the gβγ subunits of GPCRs. Activation of the PI3K/AKT survival signalling pathway inhibits apoptosis and maintains plasma membrane integrity. Moreover, the decreased intensity of mtROS/NLRP3-induced caspase-1 activation is insufficient to cleave and activate adequate amounts of GSDMD, thus preventing the formation of large pores in the plasma membrane. Chromatin decondensation during vital NETosis is independent of PAD4-mediated histone citrullination, potentially because of direct histone cleavage by mtROS or NE. The decondensed chromatin subsequently binds to granular proteins within the nucleus, forming chromatin–protein complex vesicles at the inner nuclear membrane, which are ultimately released *via* exocytosis. Following this process, the anucleated neutrophil remains viable and retains its phagocytic function. Created in https://BioRender.com. *AKT* protein kinase B, *ANCA* anti-neutrophil cytoplasmic antibody, *DAG* diacylglycerol, *FcγR* fc gamma receptor, *FoxO* forkhead box O, *GPCR* G protein–coupled receptor, *GSDMD* gasdermin D, *IP₃* inositol 1,4,5-trisphosphate, *IP₃R* inositol 1,4,5-trisphosphate receptor, *mPTP* mitochondrial permeability transition pore, mTORC1 mechanistic target of rapamycin complex 1, *mtROS* mitochondrial reactive oxygen species, *nPKC(PKCδ)* novel protein kinase C (protein kinase C delta), *N-GSDMD* N-terminal gasdermin D, *NE* neutrophil elastase, *NETs* neutrophil extracellular traps, *NETosis* neutrophil extracellular trap formation, *NLRP3* NOD-like receptor family pyrin domain containing 3, *PI3K* phosphoinositide 3-kinase, *PIP₂* phosphatidylinositol 4,5-bisphosphate, *PLCβ* phospholipase C beta, *PLCγ* phospholipase C gamma, *ROS* reactive oxygen species, *Src* Src family kinase, *Syk* spleen tyrosine kinase

Vital NETosis is characterized by the release of NETs *via* vesicular exocytosis, allowing neutrophils to retain an intact cell membrane and phagocytic capability despite becoming nonnucleated. This rapid process, occurring within minutes to 1 h, results in the formation of NETs that envelop the entire cell exterior [20, 39, 62, 87] (Figure 3). For instance, the *Staphylococcus aureus* PVL toxin induces neutrophils to release NETs in a vesicular form during migration *in vitro*. Notably, chromatin disorganization in this NET subtype does not require PAD4-mediated citrullination (H3Cit), likely because of direct histone cleavage by mtROS or NE [28]. Consequently, the disorganized chromatin binds nuclear granular proteins, forming chromatin–protein complex vesicles at the inner nuclear membrane [19, 39, 62].

The underlying mechanism involves synergistic activation of the PI3K/AKT survival signalling pathway. Complement receptor-mediated NETosis, for example, utilizes a nonclassical G protein subunit–dependent pathway [39]. Specifically, C3aR signals *via* Gαi/Gαq to promote Ca^2+^ release and PI3K activation, whereas low concentrations of C5a activate PI3K *via* the Gβγ subunits of C5aR1; activated PI3K generates PIP3 and phosphorylates AKT. Furthermore, GPCRs can engage directly the PI3K/AKT pathway through β-arrestin. This signalling axis inhibits apoptosis and maintains membrane integrity while generating insufficient downstream signals to drive robust NOX2 activation; the low levels of ROS further increase PI3K/AKT activity, thereby preventing cell death [88]. It has been suggested that the activation of MEK/ERK is not essential in this non-NOX2-dependent pathway. Furthermore, other stimuli, such as platelets, can induce NETosis by binding to neutrophil PSGL-1 *via* P-selectin, initiating the PSGL-1/Syk/Ca^2+^ axis [44, 73]. Similarly, ANCA immune complexes activate Src kinase *via* FcγR (e.g. FcγRIIa) and immobilized immune complex (iIC) signalling through FcγRIIIB [40–43, 89]. Src activation phosphorylates the ITAM domain of FcγR, recruiting and phosphorylating Syk kinase, which subsequently activates phospholipase Cγ (PLCγ). PLCγ hydrolyses PIP₂ to generate IP3 and DAG, after which IP3 triggers Ca^2+^ release from the endoplasmic reticulum [90]. Ultimately, these intracellular Ca^2+^ signals indirectly activate caspase-1 *via* mtROS/NLRP3 or calpain, leading to GSDMD cleavage and pore formation, which facilitates the release of chromatin–protein complexes through gasdermin D channels [79]. Furthermore, Src/Syk can simultaneously increase the activity of the PI3K/AKT pathway to inhibit apoptosis and maintain cell survival [90].

Although vital NETosis shares the processes of caspase-mediated cleavage and gasdermin protein activation with rapid lytic NETosis and pyroptosis, it is distinguished by the absence of membrane lysis [91–97]. As reported by Chen *et al*. [77, 78], high caspase-1 expression and caspase-11 activity drive global pore formation during rapid lytic NETosis and pyroptosis; in contrast, inflammasome assembly is restricted during vital NETosis because of the low expression of ASC and caspase-1. This results in preferential cleavage of pro-IL-1β rather than GSDMD, yielding only local membrane pores. Additionally, neutrophils may repair GSDMD pores *via* the endosomal sorting complex required for transport III (ESCRT-III) [98]. Collectively, these mechanisms preserve membrane integrity (Table 1).

In addition to GSDMD-dependent pathways, an apoptosis-driven subtype of NETosis has been described. In this process, neutrophil apoptosis acts as the upstream trigger, with caspase-3/8-mediated cleavage of GSDME (instead of GSDMD) inducing calcium influx and PAD4 activation. When efferocytosis is impaired, NET formation represents the default epigenetic programme in apoptotic neutrophils, resulting in plasma membrane permeabilization within 2–3 h and culminating in cytoplast formation. Unlike the phagocytically competent neutrophils involved in vital NETosis, these cytoplasts are membrane permeable and lack phagocytic capacity, establishing a mechanistically distinct subtype of NETosis [99].

### NETosis in the diabetic microenvironment

#### Hyperglycaemia-induced NETosis mechanisms

Activation of the MAPK/ERK and NF-κB pathways is critical for the lytic NETosis process. Prolonged hyperglycaemia also leads to the production of AGEs. The presence of a large quantity of AGEs in the microenvironment can bind to RAGE (AGER) on neutrophils, which further activates the MAPK/ERK and NF-κB pathways; this process also activates NOX2 and promotes an ROS burst, triggering lytic NETosis [35]. Furthermore, MAPK/ERK activation induces the secretion of IL-8 and TNF-α, which, in turn, leads to the recruitment of additional neutrophils. NF-κB activation increases the expression of IL-6 and IL-1β, thereby establishing a positive feedback loop that perpetuates both the inflammatory response and NETosis. NETs in the internal environment can also activate the NF-κB signalling pathway by binding to other inflammatory cells, such as TLR-4/TLR-9 on the surface of macrophages. Activated NF-κB is translocated into the cell nucleus, where it regulates the expression of a series of proinflammatory genes. For example, it upregulates the expression of NLRP3 inflammasome–related proteins and promotes the assembly and activation of the NLRP3 inflammasome, which further increases the expression of proinflammatory factors in self-inflammatory and other inflammatory cells. This forms a positive feedback loop between NETs, the NLRP3 inflammasome, and NETosis, thereby promoting the progression of inflammation [100]. Importantly, PAD4 regulates NLRP3 inflammasome assembly in neutrophils by posttranscriptionally modulating NLRP3 and ASC protein levels, establishing a bidirectional regulatory axis between PAD4 and NLRP3 that amplifies NETosis during sterile inflammation [83].

#### Metabolic reprogramming and epigenetic modifications

A sustained high-glucose microenvironment can promote the formation of NETs, and microenvironmental stresses such as hypoxia and oxidative stress localized to diabetic wounds can induce metabolic reprogramming in inflammatory cells [101–104]. This includes the activation of glycolysis and the pentose phosphate pathway (PPP), which significantly increases glycolytic flux and PPP activity through the upregulation of key glycolytic enzymes (HK2 and PFKFB3) and the rate-limiting enzyme (G6PD) of the PPP, leading to the overproduction of NADPH [105]. Recent evidence has clarified how metabolic reprogramming primes neutrophils for NET formation in the context of diabetes. Shrestha *et al*. reported that neutrophils from diabetic mice exhibit features of trained immunity, including increased glycolysis, activation of the pentose phosphate pathway, and increased fatty acid oxidation, all of which lead to substantial acetyl-CoA accumulation. ATP-citrate lyase (ACLY) mediates the production of acetyl-CoA in the nucleus, where it serves as a substrate for histone acetyltransferases (HATs) to promote the acetylation of histones H3 and H4 at residues such as H3K9, H3K14, H3K27, and H4K8. These epigenetic changes facilitate chromatin decondensation and prime neutrophils for NET release. Pharmacological inhibition of ACLY or HATs effectively blocked high glucose-induced NET priming and reversed delayed wound healing in a mouse model of diabetes [106]. Furthermore, the influx of excess pyruvate from glycolysis into mitochondria leads to electron transport chain (ETC) overload and complex I/III dysfunction, resulting in the overaccumulation of superoxide (O^2−^) [107, 108]. In addition, mtROS stabilizes the HIF-1α (hypoxia-inducible factor) protein by inhibiting prolyl hydroxylase (PHD), which, in turn, increases glycolysis [109]. This results in the formation of a glycolysis–mtROS-positive feedback loop that drives the continuous release of NETs. Similarly, a high-glucose microenvironment affects epigenetic regulation and immune memory. Chronic high-glucose exposure inhibits histone deacetylases (HDACs), leading to increased levels of acetylated histone H3K27 (H3K27ac), which opens up the promoter region of the PAD4 gene and promotes its transcription [110–112]. In addition, glucose metabolites (e.g. methylglyoxal) can directly modify histones and promote chromatin depolymerization. A high-glucose environment leads to the nonenzymatic glycosylation of histones integrated in chromatin, which can increase the proinflammatory and immunogenic properties of NETs [113–115].

#### Predominant NETosis type(s) in diabetic wounds: evidence and gaps

In the early stage of diabetic wound formation, rapid lytic NETosis induced by TLRs-TRIF or C5aR1 leads to NET formation; this process primarily serves as a rapid defence mechanism against pathogens [37–39, 54]. Diabetic wounds that are difficult to heal often develop into chronic wounds characterized by persistent inflammation caused by long-term immune microenvironment dysregulation and infection. For example, the upregulation of inflammatory factors such as IL-1β, IL-6, and TNF-α in the local microenvironment can further upregulate NOX2 through the activation of the MAPK/ERK and NF-κB pathways by TLRs/IL-1R. In addition, the accumulation of AGEs in the high-glucose microenvironment results in the overactivation of pathways such as AGEs/RAGE to provide a stable NOX2-dependent source of ROS. In contrast, the positive glycolysis-mtROS feedback loop established under chronic hyperglycaemia increases the production of mtROS, which are required for rapid lytic NETosis. In a related study, disulfiram was found to block rapid lytic NETosis by inhibiting the activation of NLRP3/Caspase-1/GSDMD in neutrophils of diabetic foot ulcer patients, thereby reducing NET formation [116]. The formation of NETs in diabetic wounds may be multimodal and parallel depending on differences among individuals and in the microenvironment and cannot be explained by a single NETosis pattern. In summary, the predominant type of NETs formed in diabetic wounds remains unclear and warrants further investigation.

### Dual roles of NETs in diabetic wound healing

#### Beneficial effects

##### Antimicrobial defence

When the body is invaded by pathogens, NETs serve as crucial components of the innate immune response. Their reticular structure, combined with various antimicrobial proteins anchored on their surface, enables effective pathogen capture. This mechanism is particularly beneficial in environments where resistance to infection is compromised, such as diabetic wounds, where it helps to limit further spread of the infection to some extent.

Notably, NETs are heterogeneous, and their functions vary significantly depending on the stimulus, pathogen type, and microenvironment. Different pathogens and external stimuli can activate the release of NETs with different functions. For example, neutrophils induced by *S. aureus* release NETs enriched in cathelicidin and protease, which can directly degrade the bacterial cell wall. Similarly, the expression of calprotectin, which can directly degrade the bacterial cell wall and inhibit bacterial metabolism *via* the chelation of zinc ions, is high in NETs released after the recognition of *Escherichia coli* by TLR4 [1, 19, 39, 62, 87]. *Mycobacterium tuberculosis* (Mtb)-induced NETs not only contain heat shock protein 72 (Hsp72), which serves as a ‘danger signal’ that triggers the release of proinflammatory cytokines from macrophages, but also specifically express cathelicidin LL-37, which targets Mtb in macrophages. Fungal β-glucans activate neutrophils to release NETs enriched in lactoferrin and pentraxin-3 *via* the Dectin-1 receptor, which binds to fungal surface polysaccharides to increase macrophage recognition [117–119]. Certain viral RNA/DNA molecules activate neutrophils *via* the RIG-I or TLR7/8 pathways, resulting in the release of NETs that integrate antiviral proteins (e.g. α-defensin and MX1) and carry mitochondrial DNA (mtDNA) to activate immune signalling [27]. Consequently, distinct pathological and microenvironmental characteristics can induce the production of ‘customized’ NETs with different functions through epigenetic modifications, posttranslational modifications, or metabolic reprogramming.

##### Repair signals at low concentrations

The beneficial effects of NETs extend beyond their antimicrobial function. At ‘physiological concentrations’, NETs can also promote wound healing by modulating the activity of other reparative cells.

Research by Chu and colleagues revealed the concentration-dependent dual role of NETs in diabetic wound healing. In contrast to high concentrations of NETs (1000 ng ml^−1^), which impair healing [120], lower concentrations of NETs (100 ng ml^−1^) preserve the function of human skin fibroblasts. Specifically, the authors demonstrated that NETs can induce the proliferation and migration of human fibroblasts by activating the CCDC25/ILK/PI3K/AKT pathway [121]. Furthermore, NETs induced by PMA significantly upregulated the expression of α-SMA, ADAM12 (a metalloproteinase), and CCN2 (a fibrogenic factor) in lung fibroblasts, promoting the differentiation of fibroblasts into myofibroblasts and increasing collagen production and migration [122]. This was demonstrated by the fact that treatment with DNase1 blocked fibroblast differentiation; however, this study did not explicitly quantify the NETs in the experimental group.

Similarly, Tonello *et al*. [123] treated HaCaT cells with PMA-induced NETs and reported the concentration-dependent bidirectional regulation of proliferation. Specifically, while low concentrations of NETs (0.05–10 ng/ml) significantly promoted HaCaT proliferation (peaking at 0.5–1 ng/ml), high concentrations (>50 ng/ml) markedly activated the TLR9-mediated NF-κB pathway, thereby inhibiting cell proliferation.

Furthermore, Aldabbous *et al*. [124] conducted established 2D/3D cultures of HPAECs from nondiabetic patients and reported that treatment with NETs, generated *via* the MPO/H₂O₂-mediated TLR4/NF-κB pathway, significantly increased endothelial tube formation and spheroid budding; upregulated the expression of multiple proangiogenic factors, including matrix metalloproteinase-9 (MMP-9), platelet-derived growth factor AB/BB (PDGF-AB/PDGF-BB), and other proangiogenic factors; and promoted endothelial cell migration. Notably, rather than using conventional mass concentrations (e.g. μg/ml), this study defined NET levels based on the relationship between neutrophil count and DNA content. Specifically, a NET DNA concentration of ~0.3 μg/ml was sufficient to trigger proinflammatory and proangiogenic responses in endothelial cells.

#### Pathological effects

In diabetic wounds, a multitude of factors, including alterations in the microenvironment, tissue damage, and local infections, increase neutrophil activation and the release of NETs [71, 125]. Moreover, in patients with diabetic wounds, excessive NETs are not confined to the local wound site but are also present in the peripheral circulation. The presence of free DNA, MPO-DNA complexes, and citrullinated histones (such as H3Cit) are considered hallmarks of the presence of NETs [31]. A plethora of experiments have demonstrated that the concentration of NETs in the peripheral blood of patients with NDU is greater than that in nondiabetic patients, while the concentration of NETs in the peripheral blood of diabetic ulcer patients, such as those with diabetic foot ulcers (DFUs), is even greater [67, 71, 126]. Consequently, excessive NET formation plays a detrimental role in the wound healing process.

##### Maintenance of chronic inflammation

Under different pathological conditions, the various components of NETs exhibit different functions. NETs can interact with immune cells, participate in immune regulation, and drive disease progression. As demonstrated in the literature, NETs in the tumour microenvironment (TIME) can activate the STAT3/STAT6 pathway. This activation is facilitated by the secretion of cytokines such as IL-8 and G-CSF, which, in turn, induce macrophages to polarize into the M2 phenotype. Additionally, NETs secrete immune inhibitory factors, including IL-10 and TGF-β. This secretion has been shown to promote tumour immune escape and metastasis [127]. In high-glucose or inflammatory microenvironments, components of NETs, such as histones and free DNA, can act as endogenous damage-associated molecular patterns (DAMPs) and be recognized by Toll-like receptors (TLRs) on macrophage surfaces. Among these, histones can be recognized by TLR-4 and TLR-9, and their unmethylated CpG motifs and phosphodiester DNA backbones can effectively activate TLR-9. Through the downstream NF-κB pathway, TLR-9 upregulates the expression of genes associated with inflammation (such as IL-8, TNF-α, IL-6, and IL-1β), which promote or maintain M1 macrophage polarization. This results in a positive feedback loop that further promotes NET formation, which prolongs the inflammatory phase of wound healing and hinders the transition to the proliferative stage [100, 127, 128]. Studies have shown that treatment with certain TLR-4 and TLR-9 antagonists can reduce the expression levels of inflammatory proteins such as NLRP3, pro-IL-1β, and IL-1β in macrophages. Furthermore, macrophages promote NET formation through exosome-mediated mechanisms. Studies have shown that when stimulated by oxidized low-density lipoprotein (oxLDL), macrophages can promote NETosis through the secretion of exosome particles containing microRNA-146a [129].

Furthermore, NETs hinder diabetic wound healing by modulating the interactions between ROS and thioredoxin-interacting protein (TXNIP). ROS play a crucial role in the activation of the NLRP3 inflammasome [130]. Indeed, nearly all agonists capable of activating the NLRP3 inflammasome require the induction of ROS. NETs have been demonstrated to increase ROS levels in macrophages. This, in turn, oxidizes thioredoxin (TRX), resulting in the release of TXNIP bound to TRX [131–133]. Upon its release, TXNIP binds to NLRP3, thereby further activating the NLRP3 inflammasome [134, 135]. This process leads to the release of inflammatory factors, such as IL-1β, which, in turn, exacerbates the inflammatory response and disrupts the normal wound healing process. Pretreatment of cells with antioxidants such as *N*-acetylcysteine (NAC) has been shown to significantly reduce ROS levels, decrease the secretion of activated caspase-1 and IL-1β, and reduce the colocalization of NLRP3 and ASC [136]. These findings highlight the importance of the ROS/TXNIP signalling pathway in mediating the effects of NETs on wound healing in the context of diabetes.

Furthermore, NETs indirectly promote inflammation by regulating T cells. Th1 and Th2 cells are the two main types of CD4+ T cells, of which Th1 cells are characterized by high levels of IL-2 and IFN-γ expression [137]. Research has demonstrated that NETs from type 1 diabetes (T1D) patients can promote the Th1 polarization of CD4+ T cells by activating dendritic cells (DCs) and can enhance macrophage-mediated inflammatory responses through the secretion of cytokines such as IL-2 and IFN-γ [138–140]. Histones and DNA in NETs can activate naive CD4+ T cells *via* TLR4. This activation results in increased mitochondrial oxidative phosphorylation (OXPHOS) [141].

##### Impaired tissue remodelling

Dysregulation of extracellular matrix (ECM) remodelling is a key feature of diabetic wounds, and emerging evidence underscores the pivotal role of the NET-MMP-9 axis in this process. Specifically, NETs released within the diabetic milieu are enriched in matrix metalloproteinase-9 (MMP-9), which actively degrades type I collagen—the structural scaffold essential for tissue repair. This excessive collagenolysis, driven by an IL-8/MMP-9-positive feedback loop *via* neutrophil–fibroblast crosstalk, creates a hostile microenvironment that directly compromises ECM integrity [142]. Consequently, despite exhibiting a profibrotic phenotype, fibroblasts in diabetic wounds fail to restore the damaged matrix, leading to impaired wound healing.

Furthermore, NETs can compromise fibroblast functionality through TLR9-mediated endoplasmic reticulum (ER) stress and apoptosis [143, 144]. Recent studies have indicated that NETs induced by a hyperglycaemic environment can trigger ER stress responses in fibroblasts, inducing the expression of stress-related proteins such as PERK, EIF2α, ATF4, and CHOP [120, 145]. ER stress disrupts the normal physiological functions of fibroblasts, which causes mitochondrial dysfunction and oxidative stress, inhibits cell proliferation and migration, and affects the synthesis of the ECM, all of which are critical for wound repair [120, 146, 147].

##### Vascular dysfunction and thrombosis

In hyperglycaemic environments, excessive neutrophil activation and NET release at wound sites may induce vascular endothelial cells (endothelial cells) to adopt proinflammatory and precoagulable phenotypes.

Crucially, the detrimental impact of the NET-MMP-9 axis, which is a key feature of the diabetic milieu, extends beyond the general ECM to the vascular endothelium [148]. Specifically, as demonstrated by Tsilingiris *et al*., NETs released in the context of type 2 diabetes are enriched in MMP-9, which actively degrades structural proteins [142]. This mechanism not only underpins the impaired tissue remodelling discussed previously but also directly compromises the integrity of the vascular basement membrane, exacerbating endothelial dysfunction. In addition, histones decorating NETs can directly or indirectly activate endothelial cell inflammatory pathways and upregulate the expression of pyroptosis-related proteins (GSDMD-N, NLRP3, and ASC), leading to the formation of pore-like structures in endothelial cell membranes and a characteristic pyroptosis-like morphology [149]. Activated endothelial cells release inflammatory factors and chemokines such as IL-8, which, in turn, recruit more neutrophils to form NETs, thereby establishing a deleterious ‘NET–inflammasome–endothelial injury’ positive feedback loop [150]. This also interferes with the eNOS (endothelial nitric oxide synthase) signalling pathway, leading to a reduction in the level of phosphorylated eNOS (p-eNOS) in endothelial cells [151].

In accordance with the findings of previous studies, NETs can inhibit the core kinases (Lats1/2 and Mst1/2) of the Hippo pathway through the TLR-9/PAK2/(Merlin/NF2 protein) pathway, leading to the dephosphorylation of YAP. YAP then binds to the transcription factor SMAD2 and translocates to the nucleus, activating the expression of genes related to endothelial–mesenchymal transition (EndMT) and thereby inhibiting angiogenesis. This process manifests as endothelial cells losing CD31 and VE-cadherin expression, acquiring mesenchymal markers (Snail-1, SLUG, and FSP-1), and exhibiting reduced migration and tubule formation capabilities [67, 152, 153]. Additionally, NETs drive EndMT by degrading VE-cadherin and subsequently activating the β-catenin signalling pathway and inducing endothelial cell death in a dose-dependent manner, with morphological changes including cell detachment and growth inhibition [154, 155]. Furthermore, endothelial cells throughout the body generally carry negative charges, which can interact with the positive charges carried by histones in NETs, making their interaction and degradation of the negatively charged glycocalyx on the surface of endothelial cells easier [149], all of which can lead to endothelial dysfunction. Additionally, S100A8/A9 (calcitonin gene–related peptides), also known as proinflammatory alarmins, are extensively secreted by neutrophils and macrophages during inflammatory states and can bind to NETs to exacerbate endothelial cell damage [156, 157].

The pathological basis for the development of DFUs lies in the peripheral vascular and neuropathic changes leading to thrombosis, which, in turn, cause local ischaemia and hypoxia at the wound site, resulting in tissue necrosis. Although the reticular structure of NETs can effectively prevent the spread of bacteria after infection, NETs can also provide binding sites for thrombotic components such as platelets, fibrinogen, and von Willebrand factor (vWF), acting as scaffolds and increasing thrombus stability [5, 158–160]. Experimental evidence has demonstrated that all thrombus components contain NETs and that histones within NETs can directly activate platelets through TLR2/4 or by binding to fibrinogen while also inhibiting protein C-thrombin modulator (TM) activity and promoting thrombin generation [161–163]. Furthermore, serine proteases such as NE and MPO have been shown to degrade the tissue factor pathway inhibitor (TFPI), thereby increasing extrinsic coagulation. In addition, these factors activate intrinsic coagulation directly by activating coagulation factor XII [164]. Activated platelets promote NETosis by releasing HMGB1 and P-selectin, thus forming a positive feedback loop [87, 158, 165–167]. Furthermore, compared with fibroblasts on the outer membrane, fibroblasts induced by NETs in thrombi exhibit high expression of TGFB1 and collagen-related genes, indicating significant differences in biological behaviour [168]. In an *in vitro* study utilizing human pulmonary arterial endothelial cells (HPAECs) from nondiabetic patients, NETs significantly increased the NFκB-dependent expression of intercellular adhesion molecule-1 (ICAM-1) in HPAECs, induced a five-fold increase in vWF expression, and substantially increased platelet adhesion to HPAECs, thus confirming the prothrombotic effects of NETs [124]. Furthermore, long-term hyperglycaemia results in the production of nonenzymatic AGEs, including glycated fibrinogen. These AGEs have been shown to increase fibrin cross-linking and codeposit with NETs, thereby further promoting thrombosis and the development of vascular lesions.

**Figure 4:**
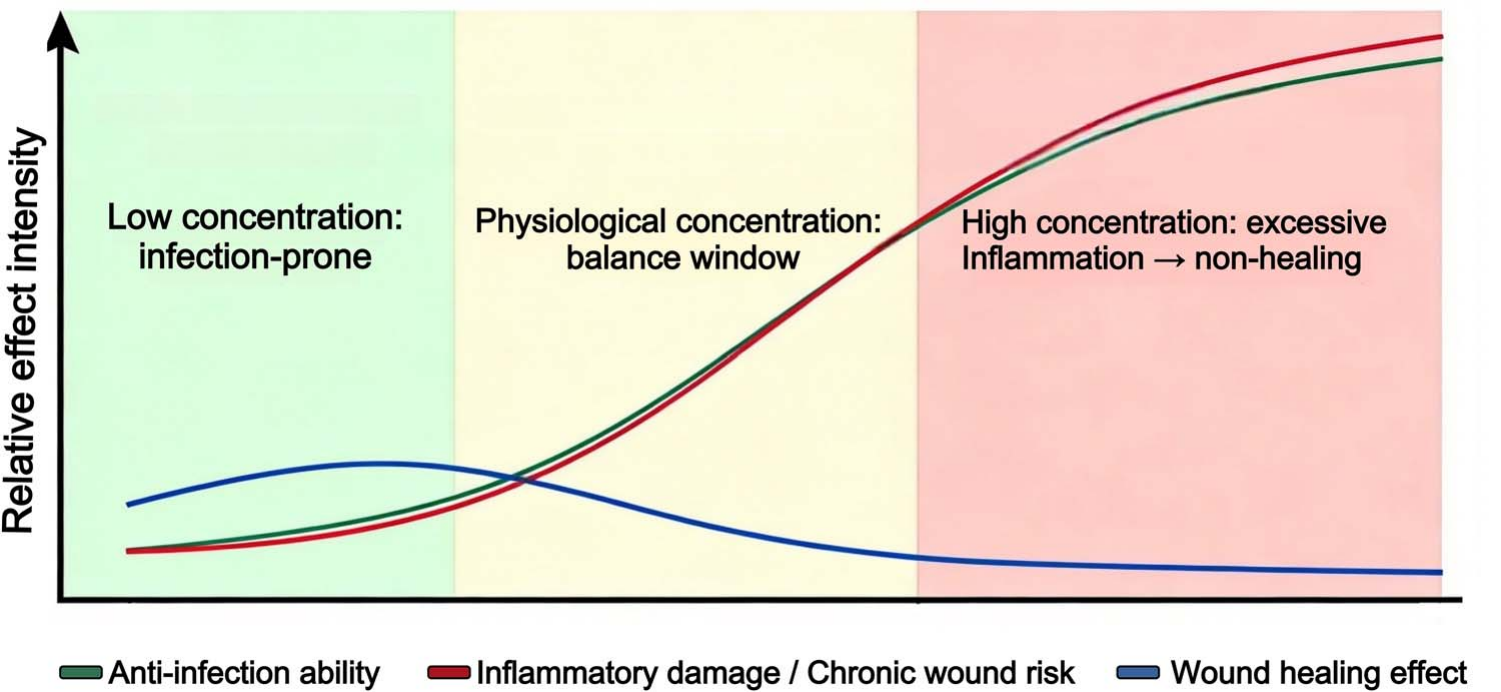
Schematic of the hypothesized relationship between NET concentration and wound healing. This diagram depicts the concentration- dependent dual effect of NETs on wound healing and defines three functional zones with increasing NET levels: Low concentration (infection-prone), physiological concentration (balanced window for optimal healing), and high supraphysiological concentration (excessive inflammation leading to wound nonhealing). Created with R 4.5.2 and ggplot2 4.0.1 [169, 170]. *NET* neutrophil extracellular trap

#### Concentration-dependent effects: towards a unified model

To date, no definitive threshold for a ‘physiological concentration’ of NETs has been established, either locally or systemically. This ambiguity is particularly consequential in wound healing, where NETs exhibit a clear dual effect that depends heavily on their concentration. At low physiological levels, NETs appear to support tissue repair, likely by assisting in pathogen clearance without inducing excessive inflammation and by promoting the repair of cells such as fibroblasts and keratinocytes. In diabetic wounds, however, excessive NET formation is normal. Although supraphysiological concentrations may increase bacterial trapping, their overall impact is detrimental, as NETs function in persistent inflammation and impair healing. However, even in controlled laboratory settings, what constitutes ‘high’ or ‘low’ NET levels varies widely across studies, and no consensus exists on a harmful versus beneficial range. Consequently, there is an urgent need to standardize NET quantification and systematically elucidate their concentration-dependent effects on wound healing. Defining an optimal concentration window (hypothesized in Figure 4) will be key to reconciling these opposing roles and guiding therapeutic strategies [169, 170].

### Therapeutic strategies targeting NETs

#### Reducing NETosis, increasing NET degradation, and neutralizing NET components

Excessive NET formation, particularly in the context of diabetic wounds, has been demonstrated to have a deleterious effect on wound healing in the overwhelming majority of cases. This has resulted in the formulation of treatment strategies that target NETs, with the primary objective being reducing NET levels in wounds and the circulation, either by decreasing NET formation or by increasing NET degradation (Table 2).

To curb NET formation at the source, interventions targeting chromatin decondensation have been extensively explored. Beyond the well-documented PAD4 inhibitors, neutrophil elastase (NE) inhibitors, such as sivelestat and GW311616A, have shown efficacy in preventing NE-mediated histone cleavage and subsequent chromatin disassembly [135, 171–179]. Additionally, neutralizing HMGB1—a critical DAMP that drives NETosis—*via* specific antibodies, glycyrrhizin, or Importin7-targeting siRNA nanoparticles (siImp7 NPs) effectively blunts the activation of downstream receptors like TLRs and RAGE [36, 180–185]. Given the indispensable role of ROS in classical NETosis, pharmacological inhibition of ROS generation using NADPH oxidase (NOX) inhibitors (e.g. DPI, tetramethylpyrazine, and VAS2870) or mitochondrion-targeted antioxidants like MitoTEMPO provides another potent avenue to block the NADPH/ROS/PAD4 axis [186–193].

Furthermore, intercepting upstream inflammatory signalling cascades offers a broader regulatory approach. Antagonists of TLR4 (e.g. TAK-242), alongside inhibitors of the TAK1, MAPK/ERK, NF-κB (e.g. BAY11–7082), and JAK2/STAT pathways, significantly attenuate the transcriptional and post-translational machinery required for NETosis [36, 51–53, 194–199]. The local immune microenvironment and cellular survival pathways can also be modulated to restrict NET release; for instance, anti-P-selectin antibodies, resveratrol, curcumin, and vitamin D exert multifaceted inhibitory effects on neutrophil activation [44, 165, 204–212]. Concurrently, targeting the CCDC25/ILK/PI3K/AKT axis has been proposed to alleviate endoplasmic reticulum stress and inadvertently suppress apoptosis-driven NET extrusion [200–203]. Metabolic reprogramming, a hallmark of diabetic neutrophils, can similarly be reversed by ATP-citrate lyase (ACLY) inhibitors to halt the epigenetic priming of NET formation [106].

Once NETs have been deployed into the wound milieu, facilitating their clearance is crucial to resolving chronic inflammation. While recombinant human DNase I directly dismantles the chromatin scaffolding of NETs [33, 66], endogenous resolution pathways can be pharmacologically augmented. Specialized pro-resolving mediators (SPMs), including resolvins, protectins, and lipoxins, not only inhibit PAD4 activity but also activate the cAMP-PKA-AMPK pathway to enhance macrophage-mediated efferocytosis and NET clearance [213–215]. Similarly, metformin has been repurposed to boost macrophage clearance of NETs while concurrently dampening neutrophil ROS production [216–219].

Finally, neutralizing the cytotoxic components of NETs, particularly extracellular histones, is vital for protecting the vascular endothelium and preventing thrombosis. Heparin neutralizes the positive charge of histones, disrupting their stable binding to DNA and causing the DNA–histone complex to dissociate [68, 122, 159, 220], whereas activated protein C (APC) directly degrades histones, thereby mitigating NET-induced endothelial pyroptosis and microvascular thrombosis [221–223]. Collectively, these multi-pronged pharmacological strategies highlight the translational potential of targeting the NET lifecycle to restore tissue homeostasis in diabetic wounds.

#### Clinical translation: current status and challenges

Although the therapeutic strategies outlined in Table 2 have shown considerable promise in preclinical settings, their translation to clinical practice remains constrained by significant gaps in evidence and methodological barriers. The current knowledge base is derived predominantly from *in vitro* systems or rodent models, which frequently fail to recapitulate the multifactorial complexity of human diabetic wounds—particularly the confounding effects of peripheral vascular disease, neuropathy, and polymicrobial infection. Furthermore, interspecies discrepancies in neutrophil biology and NETosis pathways limit the direct extrapolation of these findings to human patients, while the scarcity of long-term safety data underscores the preliminary nature of existing studies. To date, no clinical trials have specifically targeted NETosis for diabetic wound healing; however, indirect insights are emerging from the evaluation of repurposed agents such as DNase I (dornase alfa) for the treatment of chronic wounds, PAD4 inhibitors for the treatment of autoimmune disorders, and metformin for the treatment of metabolic conditions. Advancing these interventions from bench to bedside will require overcoming several critical hurdles, including the standardization of NET quantification biomarkers (e.g. MPO-DNA complexes and CitH3) for patient stratification, the development of locally delivered formulations to minimize systemic exposure, and the precise definition of therapeutic windows that balance pathological NET suppression with physiological host defences. Ultimately, successful clinical integration will likely depend on combining anti-NET strategies with established wound care protocols and emerging regenerative therapies within a biomarker-guided framework.

**Table 2:** NET-targeted treatment strategies.

**Anti-net therapeutic**	**Mechanism**	**Pharmacological component**	**Targets**	**Modes of action**	**Study type (*in vitro*/animal/clinical)**	**Ref.**
**Reduce NET formation**	**Inhibition of chromatin disassembly**	GSK484	PAD4	PAD4 is required for NETosis inhibition	*In vitro*/Animal	[67, 68]
		BB-Cl-amidine	PAD4	PAD4 is required for NETosis inhibition	*In vitro*/Animal	[64–66, 71]
		Cl-amidine	PAD4	PAD4 is required for NETosis inhibition	*In vitro*/Animal	[63, 69–71]
		GW311616A	NE	Inhibits NE-mediated histone cleavage and chromatin disassembly	*In vitro*/Animal	[135, 171–174]
		Sivelestat	NE	Inhibits NE-mediated histone cleavage and chromatin disassembly	*In vitro*/Animal	[175–179]
		Anti-HMGB1 neutralizing Abs	HMGB1	Directly binds to the HMGB1 protein, reducing the activation of the receptors TLR2, TLR4, and RAGE	*In vitro*/Animal	[180–183]
		Glycyrrhizin	HMGB1	Directly binds to the HMGB1 protein, reducing the activation of the receptors TLR2, TLR4, and RAGE	*In vitro*/Animal	[36, 184]
		Importin7-targeting siRNA nanoparticles	HMGB1	siImp7 reduces HMGB1 synthesis and secretion by blocking Importin7-mediated p38 nuclear translocation	Animal	[185]
	**Inhibition of ROS generation**	Diphenyl iodide	NOX	Inhibits NADPH/ROS/PAD4 pathway-mediated NETosis	*In vitro*/Animal	[186, 187]
		Tetramethylpyrazine	NOX	Inhibits NADPH/ROS/PAD4 pathway-mediated NETosis	Animal	[188]
		VAS2870	NOX	Inhibits NADPH/ROS/PAD4 pathway-mediated NETosis	*In vitro*	[189–191]
		Mitochondrion-targeted antioxidant	Mitochondria	Acts directly on mitochondria, targeting the removal of mitochondrial ROS	*In vitro*/animal	[192, 193]
	**Blocking inflammatory signalling pathways**	Exosomes (miR-17-5p)	Inhibits the TLR4/ROS/MAPK pathway	Inhibits intracellular ROS production	*In vitro*/animal	[120]
		TLR4 inhibitor (TAK-242)	Antagonist of TLR4 receptor	Inhibits TLR4 receptor-mediated NETosis	*In vitro*/animal	[36, 194]
		TAK1 inhibitor	MAPK pathway	Reduces ROS generation	*In vitro*/animal	[195]
		ERK inhibitor (U0126)	MAPK pathway	Reduces ROS generation, but not completely	*In vitro*/animal	[51, 196, 197]
		NF-κB inhibitor (BAY11–7082)	NF-κB pathway	Inhibits NOX2 expression and ROS generation	*In vitro*/animal	[52, 53, 198]
		JAK2 inhibitor (Ruxolitinib)	JAK/STAT pathway	Inhibits PAD4 expression	Animal	[199]
		NLRP3 inflammasome inhibitor (MCC950)	NLRP3	Inhibits NLRP3 inflammasome assembly and NETosis	*In vitro*/animal	[83]
	**Inhibition of apoptosis and NET release**	CCDC25 (coiled-coil domain containing 25)	Activation of ILK	ILK inhibits ER stress by activating the PI3K/AKT signalling pathway	*In vitro*/animal	[200–203]
	**Regulation of the immune microenvironment**	Anti-P-selectin antibody	Specifically binds to P-selectin	Inhibits PSGL-1-dependent NETosis	Animal	[44, 165, 204]
		Resveratrol	Inhibits AKT1 and RIPK1	Inhibits AKT1 and RIPK1 and reduces the release of inflammatory factors; directly reduces intracellular ROS levels	*In vitro*/animal	[205, 206]
		Curcumin	MEK/ERK pathway	Inhibits the MEK/ERK signalling pathway, downregulates PAD4 expression, and reduces NOX2 assembly	Animal	[207]
		Vitamin D	VDR/NF-κB; VDR/Nrf2 pathway	Inhibits the NF-κB pathway, reduces NOX2 expression, and downregulates proinflammatory factor expression; activates the VDR/Nrf2 pathway, increases the expression of antioxidant factors, and reduces ROS levels	*In vitro*/animal	[208–212]
	**Inhibition of metabolic reprogramming**	ACLY inhibitor (BMS303141)	ACLY	Inhibits acetyl-CoA production and histone acetylation	Animal	[106]
**Promotion of NET decomposition**	**Chromatin scaffolding for NET degradation**	Recombinant human DNase I	NET chromatin scaffolding	Directly degrades NETs	*In vitro*/animal	[33, 66]
	**Specialized pro-resolving mediators (SPMs)**	Resolvins (RvTs)	Neutrophils; macrophages	Inhibits PAD4 activity; activates the cAMP-PKA-AMPK pathway to increase macrophage-mediated NET clearance	*In vitro*/animal	[213, 214]
		Protectins	Neutrophils; macrophages	Inhibits neutrophil activation	*In vitro*/animal	[213]
		Lipoxins (Lipoxin A4)	Neutrophils	Activates the FPR2/EGR1/METTL3 signalling axis, thereby suppressing the NF-κB pathway	Animal	[215]
		Metformin	Neutrophils; macrophages	Inhibits the translocation of protein kinase C IIβ (PKC IIβ) to the cell membrane, thereby reducing ROS production; activates the cAMP-PKA-AMPK pathway to increase macrophage-mediated NET clearance	*In vitro*/animal	[216–219]
**Neutralization of NET components**	**Neutralization of the toxic components of NETs**	Heparin	Histone H3 modified on NETs	Neutralizes the positive charge of histone proteins, disrupting their stable binding to DNA and causing the DNA-histone complex to dissociate	*In vitro*/animal/clinical	[68, 122, 159, 220]
		Activates protein C	Histone H3 modified on NETs	Directly degrades histones	*In vitro*/clinical	[221–223]

*ACLY* ATP citrate lyase, *AKT* protein kinase B, *AMPK* AMP-activated protein kinase, *cAMP* cyclic adenosine monophosphate, *DNase* I deoxyribonuclease I, *EGR1* early growth response 1, *ER* endoplasmic reticulum, *FPR2* formyl peptide receptor 2, *HMGB1* high mobility group box 1, *ILK* integrin-linked kinase, *JAK2* Janus kinase 2, *MAPK* mitogen-activated protein kinase, *MEK* mitogen-activated protein kinase kinase, *METTL3* methyltransferase like 3, *MitoTEMPO* mitochondrion-targeted antioxidant, *NE* neutrophil elastase, NF-κB nuclear factor kappa B, *NLRP3* NOD-like receptor family pyrin domain containing 3, *NOX* NADPH oxidase, *Nrf2* nuclear factor erythroid 2-related factor 2, *PAD4* peptidyl arginine deiminase 4, *PI3K* phosphoinositide 3-kinase, PKA protein kinase A, *PKC IIβ* protein kinase C type II beta, *PSGL-1* P-selectin glycoprotein ligand-1, *RAGE* receptor for advanced-glycation end products, *RIPK1* receptor-interacting serine/threonine-protein kinase 1, *ROS* reactive oxygen species, *RvTs* resolvins, *SPMs* specialized pro-resolving mediators, *STAT* signal transducer and activator of transcription, *TAK1* transforming growth factor-β-activated kinase 1, *TLR2/4* toll-like receptor 2/4, *VDR* vitamin D receptor

#### Safety considerations

The primary safety concern with targeting NETs for diabetic wound therapy centres on the potential compromise of host antimicrobial defence. While excessive NETosis drives chronic inflammation in diabetic wounds and impairs their healing, NETs remain a critical component of the innate immune arsenal and are essential for pathogen containment. Systemic inhibition of NET formation could theoretically impair the clearance of bacterial and fungal pathogens, posing a significant risk given the high susceptibility of diabetic wounds to infection. Therefore, therapeutic strategies must be carefully calibrated to suppress pathological NET accumulation without abrogating essential microbial killing—a balance likely achieved through optimized local delivery systems and defined treatment windows.

Furthermore, the risk of off-target effects warrants rigorous evaluation. As the molecular machinery governing NETosis, including the PAD4, NE, and ROS signalling pathways, participates in diverse cellular processes beyond neutrophils, nonselective inhibition may disrupt vital physiological functions. For instance, PAD4-mediated histone citrullination is involved in epigenetic regulation, whereas NE plays a role in tissue remodelling. Consequently, it is imperative to develop highly specific agents and precise dosing regimens to mitigate unintended cytotoxicity and ensure a favourable risk–benefit profile in the complex metabolic milieu of diabetic patients.

## Conclusions

This review underscores the pivotal yet dualistic role of NETs in diabetic wound healing. Under physiological conditions, NETs serve as essential components of innate immunity, providing rapid antimicrobial defence and contributing to tissue repair at low concentrations. In diabetes, however, sustained hyperglycaemia, oxidative stress, AGE accumulation, and chronic infection drive pathological NET overproduction. This excessive NET burden perpetuates inflammation *via* TLR-mediated macrophage activation, disrupts tissue remodelling through fibroblast ER stress, and exacerbates vascular dysfunction by promoting endothelial-to-mesenchymal transition and thrombosis. Despite these advances, critical knowledge gaps persist. First, the predominant NETosis subtype in human diabetic wounds remains undefined, as current mechanisms rely heavily on murine or *in vitro* models that poorly recapitulate human pathophysiology. Second, no consensus exists on the quantitative threshold separating physiological from pathological NET concentrations, hindering the interpretation of their concentration-dependent effects. Third, stimulus-driven epigenetic and post-translational modifications likely dictate NET compositional heterogeneity and functional outcomes, yet this remains largely uncharacterized in diabetic wounds. Bridging these gaps requires advanced methodologies: single-cell and spatial transcriptomics can dissect neutrophil heterogeneity, map stage-specific NET-related gene expression, and uncover temporal–spatial dynamics obscured by bulk analyses. Translating preclinical anti-NET strategies into clinical practice demands careful consideration of safety, optimal delivery, and therapeutic windows, particularly given the high infection susceptibility of diabetic patients. Since wound chronicity stems from intertwined metabolic, inflammatory, and infectious drivers (e.g. bacterial biofilms), future interventions should adopt a dual-pronged strategy that concurrently targets NETs and controls infection. Clinical trials must prioritize localized delivery to minimize systemic immunosuppression and establish rigorous inclusion criteria that balance antimicrobial defence with healing promotion. Ultimately, integrating precise mechanistic insights with biomarker-guided clinical evaluation will determine whether NET-targeted therapies can transform diabetic wound management.
